# The function and morphology of Meibomian glands in patients with thyroid eye disease: a preliminary study

**DOI:** 10.1186/s12886-018-0763-9

**Published:** 2018-04-12

**Authors:** Chia-Yu Wang, Ren-Wen Ho, Po-Chiung Fang, Hun-Ju Yu, Chun-Chih Chien, Chang-Chun Hsiao, Ming-Tse Kuo

**Affiliations:** 1 Department of Ophthalmology, Taipei Tzu Chi Hospital, Buddhist Tzu Chi Medical Foundation, Taipei, Taiwan; 2 Department of Ophthalmology, Kaohsiung Chang Gung Memorial Hospital and Chang Gung University College of Medicine, Kaohsiung, Taiwan; 3 Graduate Institute of Clinical Medicine, College of Medicine, Kaohsiung Medical University, Kaohsiung, Taiwan; 4 Department of Laboratory Medicine, Kaohsiung Chang Gung Memorial Hospital and Chang Gung University College of Medicine, Kaohsiung, Taiwan; 5 Graduate Institute of Clinical Medical Sciences, Chang Gung University, Taoyuan City, Taiwan

**Keywords:** Lipid layer thickness, Meibomian gland dysfunction, Thyroid eye diseases

## Abstract

**Background:**

To investigate function and morphology of the meibomian gland (MG) in patients with thyroid eye disease (TED).

**Methods:**

In this prospective case series study, patients with unilateral or bilateral TED were consecutively enrolled. The diagnosis of TED was based on the typical orbital findings and/or radiographic evidence. The disease activity of TED was classified according to the clinical activity score (CAS). Degrees of lagophthalmos and exophthalmos, blinking rates, and results of the Schirmer test 1 were also recorded. All patients completed the SPEED questionnaire and underwent MG assessment, including lipid layer thickness (LLT), MG dropout (MGd), and MG expression.

**Results:**

In total 31 eyes from 17 patients with unilateral or bilateral TED were included. Patients were divided into inactive TED (CAS 0−1; 20 eyes from 11 patients) and active TED (CAS 2−3, 11 eyes from 6 patients) groups. MGd was significantly more severe in the active TED than the inactive TED group [Median (Inter-quartile region): 3.0 (2.0−3.0) vs. 2.0 (1.0−2.0) degree, *P* = 0.04]. However, patients with active TED had thicker LLT than those with inactive TED (90.0 [80.0−100.0] vs. 65.0 [47.8−82.5] nm, *P* = 0.02), and LLT was positively correlated with lagophthalmos (*r* = 0.37, *P* = 0.04).

**Conclusions:**

Patients with active TED had more severe MGd, but thicker LLT. Active TED may cause periglandular inflammation of MGs, leading to MGd, but compensatory secretion from residual MGs and lagophthalmos-induced forceful blinking might temporarily release more lipids over the tear film.

## Background

Thyroid eye disease (TED), also known as Graves’ ophthalmopathy and thyroid-associated orbitopathy, is an ocular manifestation of a systemic autoimmune disorder. The orbit presents the same antigens as the thyroid gland, such as the thyroid-stimulating hormone receptor, thyrotropin receptor, and insulin-like receptor [1]. Consequently, for patients with immune-related thyroid dysfunction, the circulating autoantibodies may also attack the orbit by triggering a cytokine cascade and causing orbital fibroblast proliferation, adipose tissue expansion, and glycosaminoglycan secretion [2]. Finally, patients may develop lid edema, chemosis, lid retraction, exophthalmos, lagophthalmos, restrictive myopathy, and compressive optic neuropathy, and may complain of diplopia and decreased vision [3].

Dry eye disease (DED) is very common in patients with TED: the prevalence rate of DED in TED is up to 65.2% [4, 5]. Coulter et al. reported that 97% of patients with TED in a cohort study had dry eye symptoms [6]. Some underlying mechanisms have been proposed. First, lid retraction, exophthalmos, and lagophthalmos may cause ocular surface changes and blinking abnormalities [2, 7, 8], which increase the evaporation of tears and lead to DED in patients with TED [2, 9]. Second, lacrimal acinar cells physiologically express thyroid-stimulating hormone receptors [10]; thus, the antigen−antibody reaction of TED may impair the lacrimal gland and subsequently result in a decreased volume of reflex tearing [10, 11]. Third, TED may disturb the secretion of aqueous tears and make the tear film unstable, leading to shorter tear film breakup time and increased tear film osmolality [7, 10–12].

However, increasing evidence indicates that meibomian gland (MG) dysfunction is a major risk factor of DED [13, 14]. The International Dry Eye Work Shop have classified DED into aqueous tear deficiency (ATD) and evaporative dry eye (EDE) [15, 16], and recognized MG dysfunction as the primary cause of EDE [17]. Similar to patients with MG dysfunction, patients with TED usually also have dry eye symptoms. However, previous studies only focused on the ATD in patients with TED but neglected the MG dysfunction in these patients. MGs, which are special sebaceous glands in the eyelids, secrete lipids to stabilize the tear film, decrease the surface tension, and prevent the evaporation of aqueous tears [18]. MGs are arranged in parallel palisades throughout the tarsus plates of the eyelids, and the blinking motion serves as a pumping force that releases the meibomian lipids, which are formed by meibocytes within the acini, onto the lid margin [19, 20]. TED may cause eyelid inflammation, disturb the blinking motion, and gradually change the ocular surface environment as it progresses. Therefore, we hypothesized that TED may influence the performance of MGs, similar to many systemic inflammatory diseases, such as Sjögren syndrome, psoriasis, and rosacea [21–24], causing MG dysfunction. The aim of the present preliminarily study was to investigate the performance of MGs in patients with TED.

## Methods

### Subjects

This study was a prospective case series study, which formed part of an investigation of ocular adnexal microorganisms. All procedures involving human subjects adhered to the Declaration of Helsinki. Institutional Review Board (IRB)/Ethics Committee approval was obtained from the Committee of Medical Ethics and Human Experiments of Chang Gung Memorial Hospital (CGMH), Taiwan. Informed consent was obtained from each subject in the CGMH.

Patients with hyperthyroidism and unilateral or bilateral TED were included. All participants were asked not to instill topical eye drops for 4 h and ointment for 12 h before examination. Subjects with other systemic diseases (e.g., hypertension, diabetes mellitus, connective tissue diseases, etc.), had undergone previous eyelid and ocular surgeries, or had super-active TED (clinical activity score [CAS] ≥ 4) [25] were excluded from this study. Seventeen age−sex−laterality-matched participants, who visited our clinics and met the same criteria, except for the absence of TED, were enrolled as a control group.

### Diagnosis of thyroid eye disease

Diagnosis of TED was made on the basis of 2 of the following 3 criteria [26]. First, at least 1 immune-related thyroid dysfunction (Grave’s hyperthyroidism, Hashimoto thyroiditis, circulating thyroid antibody) was present. Second, the imaging study revealed fusiform enlargement of at least 1 of the ocular muscles. Third, patients had at least 1 of the following typical orbital signs: upper eyelid retraction, exophthalmos, typical restrictive strabismus, fluctuating lid edema, or chemosis/caruncular edema [27].

Among the above ocular signs, the extent of exophthalmos was measured using Hertel’s exophthalmometer, which measures the distance of the corneal apex from the level of the lateral orbital rim [28]. The amount of incomplete or defective closure of eyelids (lagophthalmos) was measured [29]. The duration of thyroid disease, from the onset of hyperthyroidism to receiving the ocular examination of this study, was recorded.

### Classification of patients with thyroid eye disease by clinical activity score

**Fig. 1:**
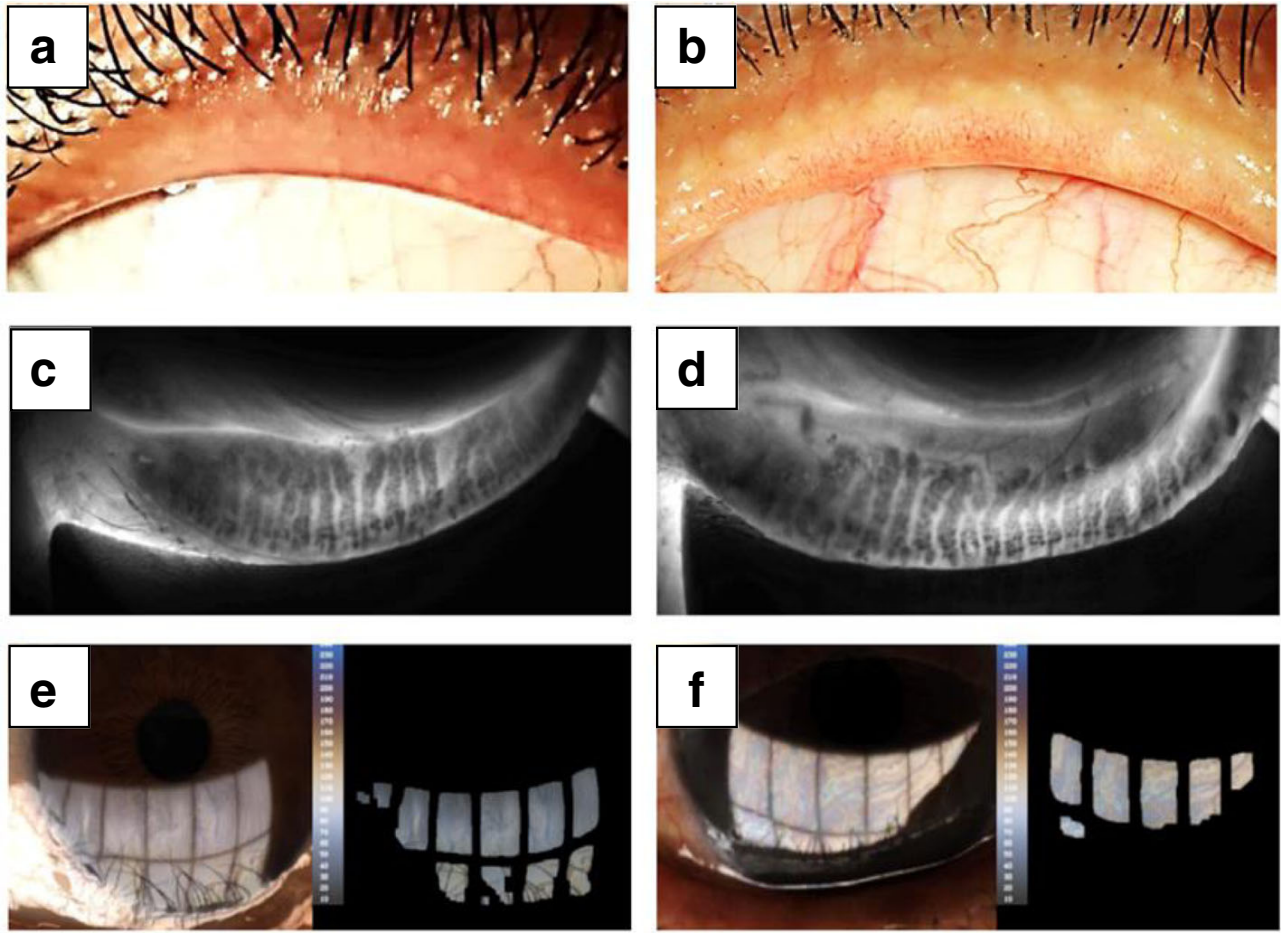
Representative photos and LipidView® II images of inactive (**a**, **c**, **e**) and active (**b**, **d**, **f**) thyroid eye diseases. **a**, **b** External eye photos of upper lid margins (OS). **a**) A 37-year-old female with inactive thyroid eye disease (CAS 1). Upper lid margin showed no pouting or capping of meibomian gland orifices. Only mild telangiectasia was observed. **b**) A 49-year-old female with active thyroid eye disease (CAS 3). Upper lid margin showed pouting and plugging of meibomian gland orifices. Telangiectasia was also present. **c**, **d** Infrared images of meibomian gland of the left lower lid. **c**) Grade 1 meibomian gland dropout (0−25%). **d**) Grade 2 meibomian gland dropout (25−50%). **e**, **f** Image of lipid layer (OS). **e**) Average lipid layer thickness: 79 nm **f**) Average lipid layer thickness: 100^+^ nm

The disease activity in patients with TED was scored according to the CAS clinical criteria proposed by Mourits et al. [25] This score contains 7 items including pain at rest, painful eye movement, red eyelid, red conjunctiva, swelling of the eyelid, chemosis, and swollen caruncle. Each item scores 1 point, so that each eye of a TED patient has a CAS score that can range from 0 to 7. For the purposes of our study, we defined inactive TED as CAS 0–1 and active TED as CAS 2–3 (Fig. 1). Super-active TED patients (CAS score ≥ 4) were excluded from this study, because the patients had unstable ocular conditions and received pulse corticosteroid therapy.

### Measurement of blinking rates and eyelid patterns

The blinking motion of the eyelids was recorded and analyzed using a LipiView® II Ocular Surface Interferometer (TearScience, Inc., Morrisville, NC, USA) [30]. Blinks without complete closure were automatically distinguished from those with complete closure by this instrument. The partial blinking rate of each eye, the ratio of incomplete blinks to total blinks within 20 s, was recorded for analysis.

### Assessment of amount of tears

Schirmer test 1 (ST-1) was used to evaluate the tear amount of each subject [31]. Without providing topical anesthesia, the Schirmer strip was suspended on the inferior eyelid between the inner two-thirds and the outer one-third for 5 min. The length of the wetting part on the test strip was then recorded for each eye.

### Standard patient evaluation of eye dryness questionnaire

The Standard Patient Evaluation of Eye Dryness (SPEED) questionnaire [32], designed for assessing the symptoms of dry eye, was used for these subjects. According to the frequency and severity of dry eye symptoms, the SPEED score ranges from 0 to 28, with asymptomatic patients scoring 0 and the most symptomatic patients scoring 28.

### Eyelid signs representing meibomian gland dysfunction

Each eye of TED patients was carefully assessed for representative signs of MG dysfunction, including plugging of MG orifices, lid margin irregularities, thickening, vascular engorgement, and mucocutaneous junction shift (Fig. 1a, b) [33, 34]. Eyes with any of the above signs were designated as MG dysfunction sign-positive.

### Grading of meibomian gland dropout

The structure of the MGs of each eye was assessed using meibography, a near-infrared (NIR) illumination captured using the LipiView® II Ocular Surface Interferometer (TearScience, Inc., Morrisville, NC). For standardization and minimization of invasiveness, only MGs in the lower eyelid were examined. The severity of MG dropout (MGd) was classified into degree 0 to degree 4 according to the meiboscale proposed by Pult et al. [35], in which the score increases by 1 degree for every 25% of MG loss (Fig. 1c, d).

### Determination of meibum quality and meibomian gland expressibility

An MG evaluator (TearScience, Inc.), which can provide a stable pressure mimicking the pressure of the orbicularis oculi muscle on MGs during normal blinking was used to press the central lower eyelid for about 10−15 s for each patient [36]. Then, the meibum quality of each gland was scored from 0 to 3 points (0: clear liquid secretion; 1: cloudy liquid secretion; 2: cloudy particulate fluid; 3: inspissated, similar to toothpaste) [37]. MG expressibility (MGE) for each patient was recorded by counting the expressible glands (glands with scores of 0, 1, or 2) from the 8 glands of the central lower lid. Scores of 0, 1, and 2 indicates that ≥5 glands, 3−4 glands, and 1−2 glands are expressible, respectively; a score of 3 indicates that no gland is expressible. MGs yielding liquid secretion (MGYLS) were also recorded by counting the glands showing liquid secretion (glands with scores 0 or 1). Moreover, a total meibum quality score (TMQS) was defined by summing the scores from the 8 glands in the central lower lid.

### Lipid layer thickness of tear film

The lipid layer thickness (LLT) of the tear film of each eye was measured and recorded using a LipiView® II Ocular Surface Interferometer (TearScience, Inc.) (Fig. 1e, f) [38]. An average interferometric color unit (ICU) (1 ICU = 1 nm) was used for quantification of LLT. Because the exact value of LLT cannot be estimated precisely or shown by this instrument if the value exceeds 100 nm, we set the LLT to 100 nm in cases where LLT ≥ 100 nm.

### Order of testing procedures

First, after each participant had completed the informed consent form, we observed the participant’s ocular surface by slit lamp, measuring the extent of exophthalmos and lagophthalmos, and recorded the SPEED questionnaire. Second, the blinking rate and LLT were simultaneously obtained by a LipiView® II Ocular Surface Interferometer, after which the structures of the MGs of the bilateral lower eyelids were sequentially assessed using the same instrument. Third, the Oxford staining score for each eye was recorded. Fourth, after each participant rested for about 30 min, ST-1 was carried out for 5 min. Finally, meibum quality and MG expressibility were determined using the MG evaluator.

### Statistical analysis

Statistical analyses were performed in SPSS version 20.0 for Windows (IBM Corp, Armonk, NY). Wilcoxon signed-rank tests and Fisher’s exact tests were used to compare the TED patients and non-TED participants. Wilcoxon’s rank-sum test was used to compare the differences of parameters between the inactive and active TED groups. Spearman’s rank correlation was used to examine the correlation between parameters. *P*-values < .05 were considered as statistically significant. Using a free power calculator (G*power; http://www.gpower.hhu.de), the sample size of at least 15 eyes was estimated based on the comparison of ST-1 and tear film break-up time between Graves’ disease patients and normal subjects according to Bruscolini A et al. [39] under the power of 0.95.

## Results

### Participants

A total of 17 patients with TED (31 eyes) were collected consecutively from the Oculoplasty Clinic of CGMH between October 2015 and June 2016. Thirty-one age−sex−laterality-matched non-TED eyes were consecutively enrolled for comparison. The clinical profiles of these subjects are summarized in Table 1. When comparing the TED with the non-TED group, the indices of TED complications, including degrees of exophthalmos and lagophthalmos, were significantly different between the groups. However, there was no statistically significant difference in partial blinking rate, tear volume, or SPEED score. Moreover, among the indices of MG performance, only MG dysfunction signs and MGYLS showed statistically significantly differences between TED and non-TED eyes.

**Table 1:** The clinical characteristics of the study subjects

	TED group (31 eyes of 17 patients)	Non-TED group (31 eyes of 17 controls)	*P* value^†^
Age (yr)	44.7 ± 11.0	44.7 ± 11.2	0.785
CAS	1.6 ± 0.7	–	–
Disease duration (months)	47.3 ± 63.5	–	–
Exophthalmos (mm)	20.4 ± 4.3	16.5 ± 1.1	< 0.001^*^
Lagophthalmos (mm)	0.9 ± 1.9	0.0 ± 0.0	0.015^*^
Partial blinking rate (%)	58.4 ± 34.7	55.1 ± 34.1	0.702
ST-1 (mm)	12.6 ± 9.0	13.0 ± 10.2	0.885
SPEED (score)	7.4 ± 4.1	6.8 ± 4.7	0.509
MG dysfunction signs^‡^ (eyes)	27	1	< 0.001^*^
MGd (score)	2.1 ± 0.9	2.2 ± 0.9	0.555
MGE (score)	0.6 ± 1.1	0.8 ± 0.9	0.379
MGYLS (score)	4.7 ± 3.1	3.0 ± 2.5	0.014^*^
TMQS (score)	10.1 ± 8.0	8.5 ± 4.9	0.412
LLT (nm)	74.1 ± 21.7	72.0 ± 21.9	0.654

†Fisher’s exact test was used to test the between-group differences in MG dysfunction signs, while the Wilcoxon signed-rank test was used to test other parameters

(*) Statistically significant (*P* < 0.05)

‡MG dysfunction signs, signs of meibomian gland (MG) dysfunction, including irregular lid margin, vascular engorgement, plugged meibomian gland orifices, and displacement of mucocutaneous junction

*CAS* Clinical activity score, *ST-1* Schirmer test 1, *SPEED* Scores of SPEED questionnaire, *MGd* MG dropout, *MGE* MG expressibility, *MGYLS* MG yielding liquid secretion, *TMQS* Total meibum quality score, *LLT* Lipid layer thickness

### Comparison between active and inactive TED from the indices of TED complications

When we compared the active and inactive TED eyes, the degrees of exophthalmos and lagophthalmos were significantly higher in active TED eyes (*P* = .01 and *P* < .001, respectively), while the partial blinking rate and ST-1 were not statistically significantly different (*P* = .83 and *P* = .20, respectively). Moreover, there was no significant difference in the SPEED score between the active and inactive TED patients (*P* = .80) (Table 2).

**Table 2:** Comparison between active and inactive thyroid eye diseases

	Active TED^†^ (11 eyes of 6 patients)	Inactive TED^‡^ (20 eyes of 11 patients)	*P* value^§^
Age (yr)	45.2 ± 16.1	44.4 ± 7.3	0.679
Sex (female: male)	7: 4	11: 9	0.718
Disease duration (months)	80.8 ± 96.8	28.9 ± 20.5	0.264
Exophthalmos (mm)	23.1 ± 4.3	18.5 ± 3.3	0.005^*^
Lagophthalmos (mm)	2.13 ± 2.67	0.18 ± 0.59	< 0.001^*^
Partial blinking rate (%)	58.2 ± 29.9	58.5 ± 37.8	0.834
ST-1 (mm)	9.2 ± 5.6	14.5 ± 10.0	0.199
SPEED (score)	7.1 ± 4.6	7.6 ± 3.9	0.803
MG dysfunction signs^#^ (no. of eyes; %)	11 (100%)	16 (80%)	0.269
MGd (score)	2.5 ± 0.9	1.8 ± 0.7	0.03^*^
MGE (score)	0.2 ± 0.4	0.9 ± 1.2	0.148
MGYLS (score)	5.4 ± 2.9	4.3 ± 3.1	0.362
TMQS (score)	8.2 ± 5.5	11.2 ± 9.0	0.482
LLT (nm)	86.3 ± 18.0	67.4 ± 21.0	0.024^*^

^†,‡^Patients with thyroid eye diseases (TED) were classified by the clinical activity score (CAS) as having active TED (CAS 2−3) or inactive TED (CAS 0−1); ^§^Fisher’s exact test was used to test the between-group differences in sex and MG dysfunction signs, while Wilcoxon’s rank-sum test was used to test other parameters. (^*^)Statistically significant (*P* < 0.05)

^#^MG dysfunction signs, signs of meibomian gland (MG) dysfunction, including irregular lid margin, vascular engorgement, plugged meibomian gland orifices, and displacement of mucocutaneous junction

### MG performance in active and inactive TED eyes

**Fig. 2:**
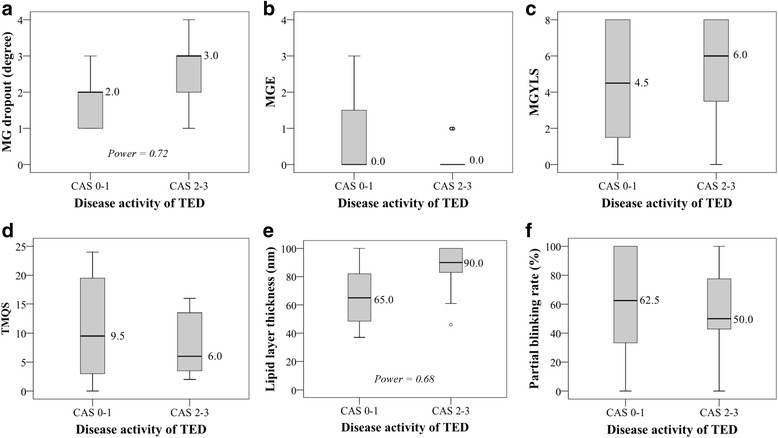
Comparison between active and inactive thyroid eye diseases (TED) for meibomian gland dropout (MGd), meibomian gland expression, including meibomian gland expressibility (MGE), meibomian gland yielding liquid secretion (MGYLS), and total meibum quality score (TMQS), as well as lipid layer thickness (LLT), and partial blinking rate, by boxplot diagrams. The dot in Fig. 2b shows the 2 outliers of MGE in CAS 2−3. Box, 25th to 75th percentile; bold line in the box, median; bars, minimum and maximum values; dot, outliers

Among the parameters of MG performance, MGd and LLT were statistically significantly different between active and inactive TED eyes. MGd was significantly more severe in active TED eyes than in inactive TED eyes (*P* = .03). However, in conflict with the inference from the MGd result, active TED eyes had significantly thicker LLT than did those with inactive TED (*P* = .02). There were high proportions of TED patients with MG dysfunction signs. All active TED eyes and up to 80% of inactive TED eyes had signs of MG dysfunction, but there was no statistically significant difference between the 2 groups (*P* = .27). Moreover, the 2 groups did not show significant differences in MG expression, including MGE (*P* = .15), MGYLS (*P* = .36), and TMQS (*P* = .48) (Table 2 and Fig. 2).

### Correlation between MG performance and TED complications

**Fig. 3:**
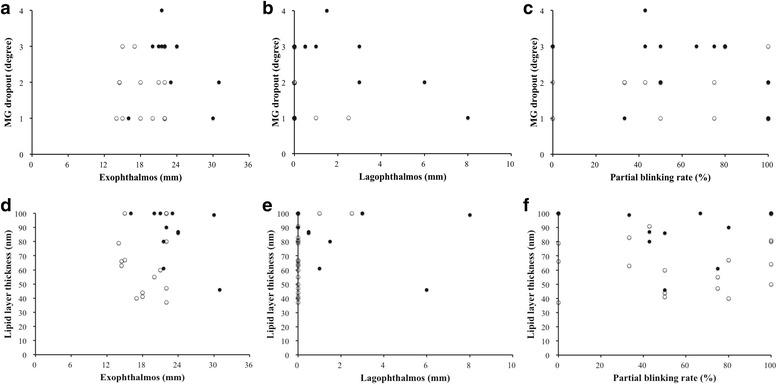
Correlation between meibomian gland dropout (MGd) and exophthalmos, lagophthalmos, and blinking rate, respectively (**a**−**c**). Correlation between lipid layer thickness (LLT) and exophthalmos, lagophthalmos, and blinking rate, respectively (**d**−**f**). (○), inactive TED eye (CAS 0−1); (●), active TED eye (CAS 2−3)

To investigate the impact of TED activity on MG performance, a correlation analysis between the parameters of MG performance and the indices of TED complications was performed. There was no significant association between MGd and TED complications, including exophthalmos, lagophthalmos, and partial blinking rate (Fig. 3). However, a significantly positive correlation was found between LLT and lagophthalmos (*r* = 0.37, *P* = .04), but no other parameters of TED complications correlated with LLT.

## Discussion

The impact of MG dysfunction, the leading cause of DED, on patients with TED, has remained unclear. Interestingly, 27 eyes (87.1%) had signs of MG dysfunction, but the mean age of these TED patients was only 44.7 years (Table 1). We also found that active TED eyes (CAS 2−3) had a higher MGd, but thicker LLT, than inactive TED eyes (CAS 0−1) (Table 2 and Fig. 2). Although active TED eyes had more severe exophthalmos and lagophthalmos, only lagophthalmos was associated with a thicker LLT (Fig. 3). The finding of a thicker LLT, but higher MGd, in active TED suggests not only compensatory activity from the residual MGs, but also that lagophthalmos-mediated forceful blinking is involved. This could be a potential mechanism for decreasing ocular surface injury from the more severe lagophthalmos in active TED.

Active ocular inflammation may cause typical ocular surface changes in TED in patients with thyroid disease [2]. We hypothesized that active TED would further impede the performance of the MGs in these patients. Although the CAS score proposed by Mourits to reflect the inflammation status of TED ranged from 0 to 7 [25], only patients with a CAS of less than 4 were included in this study. All patients with CAS exceeding 3 received pulse-corticosteroid treatment, and they were excluded from this study to prevent a possible bias due to this treatment. A flair-up of TED may result in greater extraocular muscle enlargement, and this may be reflected in the greater exophthalmos and lagophthalmos of the active TED eyes in this study (Table 2).

In this study, the mean MGd of TED patients was 2.1 (Table 1), representing about 25−50% loss of MGs. Active TED eyes had significantly greater loss of MGs than inactive TED eyes (Table 2). However, loss of MGs was not associated with the target indices of TED complications (Figs. 3a, b, and c). Several studies have shown a strong association between MGd and inflammatory ocular surface diseases. Mathers et al. reported that patients with chronic blepharitis and giant papillary conjunctivitis demonstrated a greater loss of MGs [40, 41]. Shimazaki et al. reported that Sjögren syndrome was associated with MG dysfunction [22]. Knop et al. pointed out that inflammatory mediators could spread and lead to glandular dropout and potentially to acinar atrophy by way of the conjunctiva, through the tarsus and toward the MGs [42]. Thus, TED-associated ocular surface inflammation might cause periglandular inflammation, and subsequent loss of MGs.

A recent study proposed that a thinner LLT may predict a higher risk of MG dysfunction [43]. Eom et al. also found that greater loss of MGs is correlated with a thinner LLT [44]. It is reasonable that a stasis of lipid inside the MGs may increase pressure in the MGs, causing the ducts to dilate, and finally resulting in acinar atrophy [19]. However, in our study, the active TED eyes showed a greater loss of MGs, but thicker LLT, than the inactive TED eyes (Table 2 and Fig. 2). Additionally, we found a positive correlation between lagophthalmos and LLT (Fig. 3), and active TED eyes had greater lagophthalmos than inactive TED eyes (Table 2). Kim et al. reported that some MGs may be obstructive and atrophic, while other MGs may secrete lipids at normal or enhanced levels to compensate for MG dysfunction, whereby normal LLT is maintained [45]. Korb et al. found that forceful blinking could increase the LLT [46]. It is possible that active TED patients had more severe MGd, but thicker LLT, not only as a compensatory effect, but also as a stimulatory effect. The compensatory effect may be induced by MGd, based to some degree on a physiological response from residual MGs, but not on the over-production of lipids. However, patients with more severe lagophthalmos may show more severe punctate erosion on the ocular surface. Additionally, MG disease may increase corneal sensitivity [47]. Therefore, active TED patients with more severe lagophthalmos may have pathological lipid hypersecretion due to forceful blinks. Patients with TED might blink more forcefully, unconsciously, due to greater lagophthalmos. Although the active TED eyes demonstrated more severe MGd, the mixed compensatory and stimulatory effect may cause temporarily thicker LLT than that seen in inactive TED eyes.

In addition, the function of MGs may be maintained at a certain level, because the loss of MGs is partial (on average 25−50%) even in active TED eyes. Active TED eyes had greater exophthalmos than inactive TED eyes (Table 2 and Fig. 2). Exophthalmos could stretch the eyelid, inducing even higher lid tension and making it easier for the lipids in the MGs to be squeezed out. However, there was no significant correlation between exophthalmos and LLT (Fig. 3).

All but 1 of the patients with TED suffered from dry eye symptoms, as identified on the SPEED questionnaire. Despite the lack of statistically significant difference, lower aqueous tear secretion (ST-1) was found in patients with active TED (9.2 ± 5.6) than in patients with inactive TED and in the normal control group (14.5 ± 10.0 and 13.0 ± 10.2, respectively). These findings were compatible with those of Eckstein et al. [10], who concluded that ATD may be caused by diminished lacrimal gland function in active TED. The expression of inactive TED may be the same as proposed by Arita et al. [48], who pointed out that increased tear fluid is produced as a temporary compensatory response to loss of MGs.

There were some limitations in this preliminary study. The non-TED control group may be suitable for comparison with the TED group in our clinical practice, but this control group cannot truly represent a normal population. Some participants in the control group also had dry eye symptoms and inadequate MG performance. Both eyes of bilateral TED patients were pooled with the 3 eyes of the 3 unilateral TED patients. We had found similar results in the analyses of right eyes or left eyes. Although a trend for thicker LLT in active TED was noted, the wide range of standard deviation resulted in a non-significant difference. Thus, we classified both eyes of the same patient into the same CAS group, which might have caused a bias. However, all patients with active TED (CAS 2−3) in our study had ocular signs of similar severity, only 2 patients with inactive TED (CAS 0−1) had single eye involvement. One eye in the active TED group was excluded due to previous eyelid surgery. Furthermore, most patients routinely used eye ointment for lubrication at night. The usage of topical ointment should be more strictly limited to avoid its influence on LLT. The ingredients of an eye ointment might affect the tear film composition, yet all patients had ceased ointment application at least 12 h before examination. Moreover, we set LLT as 100 nm for the 5 eyes with LLT > 100 nm, which may have caused underestimation of the average value of LLT. Because the LipiView® II Ocular Surface Interferometer did not have a sensor to identify the blinking force, we cannot clearly prove the association between LLT and forceful blinks. A further study adopting simultaneous electromyography of the eyelid should be considered to verify this causality. Finally, the small sample size implies that our results should be interpreted with some caution. Subgroup analysis revealed that inactive TED eyes were not significantly different from non-TED eyes in terms of MG performance and LLT, but a trend for greater MGd and thicker LLT was observed between active TED eyes and non-TED eyes. Thus, a small proportion of active TED patients in our subjects might be the reason for the lack of statistically significant differences in many parameters between the TED group and the non-TED group. The performance of MGs in TED patients should be verified in a future study with a larger sample.

## Conclusions

In conclusion, the results of this study indicated that patients with active TED had more severe MGd, but thicker LLT. Active TED may cause periglandular inflammation of MGs, leading to MGd, although lagophthalmos might induce a compensatory effect, involving increased lipid secretion from the residual MGs in an attempt to stabilize the tear film.

## Abbreviations

ATD: Aqueous tear deficiency
CAS: Clinical activity score
DED: Dry eye disease
EDE: Evaporative dry eye
LLT: Lipid layer thickness
MG: Meibomian gland
MGd: Meibomian gland dropout
MGE: Meibomian gland expressibility
MGYLS: Meibomian gland yielding liquid secretion
SPEED: Standard patient evaluation of eye dryness
ST-1: Schirmer test 1
TED: Thyroid eye disease
TMQS: Total meibum quality score
